# A simple strategy to prepare a layer-by-layer assembled composite of Ni–Co LDHs on polypyrrole/rGO for a high specific capacitance supercapacitor

**DOI:** 10.1039/c9ra08134h

**Published:** 2019-12-06

**Authors:** Shalini Kulandaivalu, Mohd Zobir Hussein, Adila Mohamad Jaafar, Muhammad Amirul Aizat Mohd Abdah, Nur Hawa Nabilah Azman, Yusran Sulaiman

**Affiliations:** Department of Chemistry, Faculty of Science, Universiti Putra Malaysia 43400 UPM Serdang Selangor Malaysia yusran@upm.edu.my +603-89435380 +603-89466779; Materials Synthesis and Characterization Laboratory (MSCL), Institute of Advanced Technology (ITMA), Universiti Putra Malaysia 43400 Serdang Selangor Malaysia; Centre of Foundation Studies for Agricultural Science, Universiti Putra Malaysia 43400 Serdang Selangor Malaysia; Functional Devices Laboratory, Institute of Advanced Technology, Universiti Putra Malaysia 43400 Serdang Selangor Malaysia

## Abstract

A facile and novel electrode material of nickel–cobalt layered double hydroxides (Ni–Co LDHs) layered on polypyrrole/reduced graphene oxide (PPy/rGO) is fabricated for a symmetrical supercapacitor *via* chemical polymerization, hydrothermal and vacuum filtration. This conscientiously layered composition is free from any binder or surfactants which is highly favorable for supercapacitors. The PPy/rGO serves as an ideal backbone for Ni–Co LDHs to form a free-standing electrode for a high-performance supercapacitor and enhanced the overall structural stability of the film. The well-designed layered nanostructures and high electrochemical activity from the hexagonal-flakes like Ni–Co LDHs provide large electroactive sites for the charge storage process. The specific capacitance (1018 F g^−1^ at 10 mV s^−1^) and specific energy (46.5 W h kg^−1^ at 464.9 W kg^−1^) obtained for the PPy/rGO|Ni–Co LDHs symmetrical electrode in the current study are the best reported for the two-electrode system for PPy- and LDHs-based composites. The outstanding performance in the prepared LBL film is a result of the LBL architecture of the film and the combined effect of redox reaction and electrical double layer capacitance.

## Abstract 2

A facile and novel electrode material of nickel–cobalt layered double hydroxides (Ni–Co LDHs) layered on polypyrrole/reduced graphene oxide (PPy/rGO) is fabricated for a symmetrical supercapacitor *via* chemical polymerization, hydrothermal and vacuum filtration.

## Graphical abstract

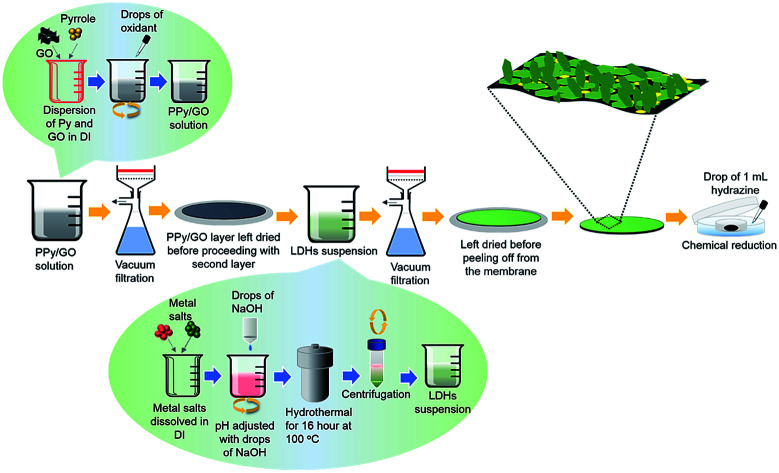

## 1. Introduction

As the need for energy storage devices in this modern world continues to develop extensively, the demand for lightweight, durable, portable and flexible devices with a longer life cycle, higher specific energy and specific power becomes ubiquitous. The evolution of electrochemical supercapacitors (SCs) has become an effective strategy to acquire next-generation energy storage devices with the abovementioned qualities and it is also expected to replace batteries and conventional capacitors in the future. SCs are made up of a simple configuration consisting of current collectors, an electrolyte, a separator and active materials. Each component plays a pivotal role in providing better performing SCs. However, active materials are considered as the core component of high performance SCs.

Pseudocapacitive materials (conducting polymers and metal oxides/hydroxides) have shown to be SC electrodes with high specific capacitance. However, they usually suffer from poor stability performances.^6,7^ On the other hand, the commonly employed carbon-based materials, for instance, graphene oxide (GO) and reduced graphene oxide (rGO) are the types of electrical double layer capacitive materials known for their extraordinary life cycles and high electrical conductivity.^8^ However, due to the poor specific capacitance of GO and rGO, these materials are often combined with pseudocapacitive materials for SCs. By combining the distinctive features of each pseudocapacitive and electrical double layer capacitive materials, the emergence of hybrid materials as active materials have led to inspiring opportunities to enhance the performance of SCs. Polypyrrole (PPy) is being the most explored conducting polymer that usually combined with carbon-based materials namely GO and rGO to improve its properties.^9,10^

Among the metal oxides/hydroxides, layered double hydroxides (LDHs) particularly nickel–cobalt LDHs (Ni–Co LDHs) are an emerging electrode material for SCs due to its high theoretical specific capacitance owing from synergistic effects of its two cations.^11^ Additionally, Ni–Co LDHs also have a high specific area, low cost, environment friendliness and its layered structure allow faster ion intercalation–deintercalation process.^1^ However, similar to conducting polymers, Ni–Co LDHs have a lack of structural stabilities under continuous charge–discharge process. Therefore, in recent years, LDHs often combined with graphene-based materials. Wang, *et al.*^12^ have prepared 3D hierarchical porous Ni–Co LDHs/nitrogen-doped rGO composite in an asymmetrical SC with a specific capacitance of 100 F g^−1^ at 0.5 A g^−1^ and stability retention of 83% over 10 000 cycles. In another study, Mehrabimatin, *et al.*^11^ have fabricated Ni–Co LDHs/nitrogen-doped rGO on carbon cloth in the asymmetrical assembly which delivered specific capacitance of 109 F g^−1^ at 0.5 A g^−1^ and stability retention of 82% over 2000 cycles.

A layer-by-layer (LBL) assembly is a possible way to hybrid different carbon materials, metals oxides and metal hydroxides in a single composite whilst maintaining its properties. LBL assembly is identified as a suitable approach in producing active materials for SCs due to a simple and straightforward approach to produce a nanostructured material with multilayer composition by having interactions/forces between the layers with controlled composition and structure.^13^ There are few studies conducted on LBL assembly in order to prepare the electrode materials for SCs and interestingly those studies disclosed extraordinary electrochemical performances.^10,14,15^

Therefore, herein, we reported a facile fabrication of Ni–Co LDHs *via* hydrothermal and for the first time, layered it on PPy/GO (PPy/GO|Ni–Co LDHs) by utilizing the LBL assembly concept through a simple vacuum filtration method. The PPy/GO serves as a support for Ni–Co LDHs, where the LDHs anchored firmly on the PPy/GO layer. Subsequently, the prepared LBL film was subjected to chemical reduction through the hydrazine vapor method to form PPy/rGO|Ni–Co LDHs film. The resultant films showed extraordinary electrochemical performances due to the synergistic effects of multiple components in the film. Furthermore, a few other factors also need to take into point, (i) the absence of binder in the electrode fabrication which can hinder the electrical resistance from the binder, (ii) the well-defined nanoflake-like structure of Ni–Co LDHs attached on PPy/rGO advances the ion mobility providing short pathway, and (iii) introduction of Ni–Co LDHs on PPy/rGO provides an easily accessible channel to electrolytes. As a symmetrical SC active material, the PPy/rGO|Ni–Co LDHs LBL film revealed high specific capacitance and specific energy with desirable capacitance retention making it suitable electrode material for SCs.

## 2. Experimental

### 2.1 Materials/chemical

Graphene oxide (GO) was purchased from Graphenea. Pyrrole monomer (Py; 97%) was obtained from Merck and distilled prior to use. Nickel nitrate hexahydrate (Ni(NO_3_)_2_·6H_2_O; 98%) and cobalt nitrate hexahydrate (Co(NO_3_)_2_·6H_2_O; 98%) were supplied by Sigma Aldrich. The reducing agent, hydrazine monohydrate (N_2_H_4_·H_2_O, 100%) was supplied by Nacalai Tesque, Inc. Ferric chloride hexahydrate (FeCl_3_·6H_2_O, 98%) employed as an oxidizing agent was procured from Bendosen. Sodium hydroxide (NaOH; 98.4%) and sodium sulfate (Na_2_SO_4_; 99%) were obtained from Friendemann Schmidt and Merck, respectively. Indium tin oxide (ITO) coated glass with a sheet resistance of 7 Ω sq^−1^ acquired from Xin Yan Technology Limited was utilized as a current collector. The cellulose nitrate membrane filters (pore size, 0.45 μm and *∅*, 47 mm) and filter papers were received from GE Healthcare Life Science, UK and Whatman, UK, respectively. Deionized water (Millipore Milli-Q, 18.3 MΩ cm @ 25 °C) was used throughout the experimental. All chemicals utilized in this study were of analytical grade and used without any further purification unless otherwise stated.

### 2.2 Preparation of polypyrrole/reduced graphene oxide and layered double hydroxide precursor

The PPy/GO solution was prepared *via* a simple and facile *in situ* polymerization method. Prior to synthesis, the starting material, aqueous GO dispersion (1 mg mL^−1^) was ultrasonicated for 60 min to produce a homogenous and stable GO suspension. 100 mM Py monomer was then added to the suspension. After a constant stirring for 15 min, 100 mM FeCl_3_·6H_2_O was added dropwise into the Py/GO mixture. The polymerization was allowed to proceed for 24 h at room temperature under constant stirring to produce PPy/GO solution.

Ni–Co LDHs suspension was prepared through a hydrothermal. Firstly, a transparent pink solution was prepared by mixing Ni(NO_3_)_2_·6H_2_O and Co(NO_3_)_2_·6H_2_O metal salts in a molar ratio of 2 : 1 in 100 mL deionized water. The mixture was then stirred for 15 min before adjusting the pH of the mixture by adding 2 M NaOH slowly under a vigorous stirring and nitrogen gas flow. Subsequently, the resultant solution mixture was transferred to a Teflon-lined stainless-steel autoclave. The Teflon-lined autoclave was sealed and kept to 100 °C for 16 h to allow the growth of Ni–Co LDHs. After cooling to room temperature, the obtained LDHs slurry was washed repeatedly with deionized water by centrifugation for 5 min at 4500 rpm and then re-dispersed in deionized water. For comparison, Ni–Co LDHs powder was also prepared in a similar procedure where after centrifugation, the green precipitates were collected and dried overnight at 60 °C.

### 2.3 Preparation of layer-by-layer assembled composite films and fabrication of symmetrical electrode

**Fig. 1:**
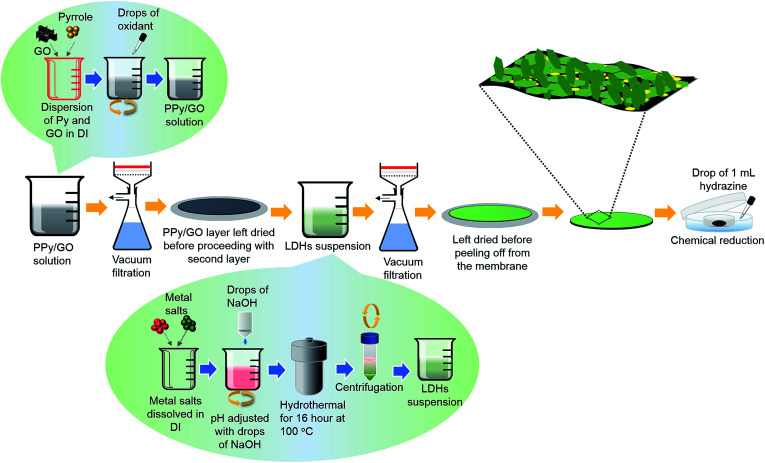
Schematic illustration of the preparation of LBL film comprising chemical polymerization, hydrothermal process and vacuum filtration.

The schematic diagram of the preparation of LBL composite film is illustrated in Fig. 1. PPy/GO solution (5 mL) was vacuum filtered through a membrane filter to obtain a PPy/GO layer and was left to dry for a few minutes.^10^ Next, Ni–Co LDHs suspension (8 mL) was vacuum filtered onto the PPy/GO layer. After drying for 4 h at 60 °C, the LBL film was carefully peeled off from the membrane filter forming a free-standing PPy/GO|Ni–Co LDHs film. As-fabricated LBL film was then subjected to chemical reduction *via* a modified hydrazine vapor method at 45 °C for 18 h.^10^ Two rectangle pieces (1 cm^2^) of the formed LBL film were then arranged into a two-electrode configuration separated by a piece of filter paper soaked in 1 M Na_2_SO_4_. The whole assembly was then sandwiched between two pieces of ITO. The average weight of the active materials was ∼0.25 mg.

For a comparison purpose, PPy/rGO (4 mL) and rGO|Ni–Co LDHs symmetrical SCs were also fabricated using the same procedures.

### 2.4 Material characterization and electrochemical measurements

Field emission scanning electron microscope (FESEM, JEOL JSM-7600F) operated at an acceleration voltage of 5.0 kV combined with energy dispersive X-ray spectroscopy (EDX) was utilized to study the morphology and composition of the prepared films. Fourier transform infrared spectra (FTIR) spectra were recorded *via* Shimadzu FTIR spectrometer. Raman spectra were acquired by Alpha300 R microscopic confocal Raman spectrometer (WITec GmbH) using an excitation source of 532 nm. X-ray diffraction (XRD) patterns were collected from Shimadzu X-ray diffractometer with Cu Kα radiation (*λ* = 1.54 Å).

All the electrochemical measurements were conducted in a two-electrode symmetrical SC assembly on a potentiostat/galvanostat (Autolab 101) electrochemical workstation equipped with NOVA software. The cyclic voltammetry (CV) curves were recorded in a potential window of 0 to 1 V at different scan rates (10 to 200 mV s^−1^). The galvanostatic charge–discharge (GCD) tests were conducted within the potential range of 0 to 1 V at current densities ranging from 0.5 to 5 A g^−1^. The electrochemical impedance spectroscopy (EIS) data was collected within the frequency range of 10 mHz to 100 kHz with a sinusoidal perturbation amplitude of 5 mV at open circuit potential. All the experiments were carried out at room temperature.

## 3. Results and discussion

### 3.1 Field emission electron microscopy

**Fig. 2:**
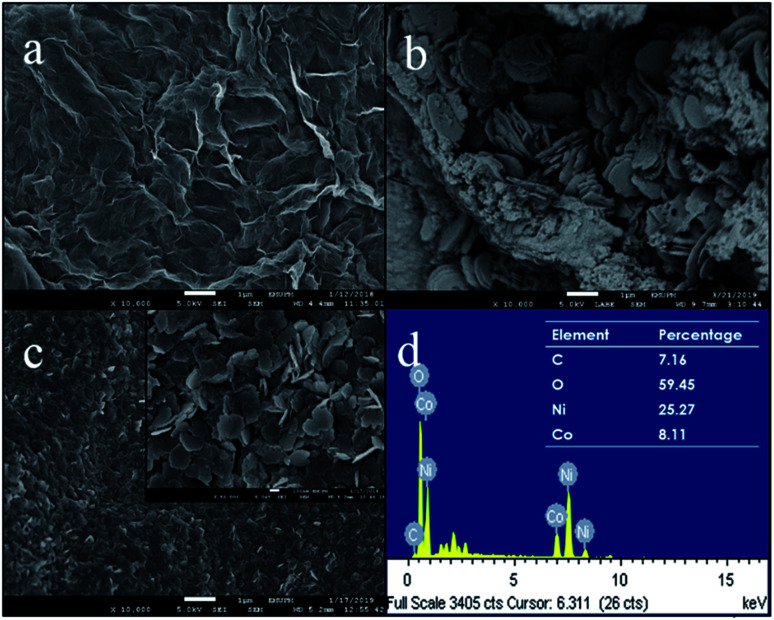
FESEM images of (a) PPy/rGO film, (b) pure Ni–Co LDHs powder, (c) PPy/rGO|Ni–Co LDHs LBL film (inset shows higher magnification FESEM image) and (d) EDX pattern of LBL film (inset shows the detailed elemental composition).

The surface morphologies of PPy/rGO film, pure Ni–Co LDHs powder and PPy/rGO|Ni–Co LDHs LBL film were investigated *via* FESEM measurements and the micrographs are shown in Fig. 2. The FESEM micrograph of the PPy/rGO film shows a wrinkled surface texture resembling crumpled sheets of rGO and the PPy is uniformly covered within the rGO sheets. Moreover, the heavily folded wrinkles indicate strong van der Waals forces^16^ between the sheets, and it should be pointed out that there is no aggregation found in the composite. The micrograph of pure Ni–Co LDHs (Fig. 2b) reveals non-uniform hexagonal flakes. The size of flakes is in the range of 1.3 to 1.5 μm and the flakes are mostly overlapped or stacked on top of each other. Therefore, a dense structure can be noticed. On top of that, the dried Ni–Co LDHs powder exhibits aggregated particles in certain areas. Whereas, after the formation of LBL film, a more uniform distribution of LDHs flakes is observed (Fig. 2c). The existence of the Ni–Co LDHs on the PPy/rGO is also supported by EDX analysis by detecting Ni, Co and O elements (Fig. 2d). The magnified view of LBL film further discloses that the flakes still remain in hexagonal plate-like shapes. The hexagonal flakes are randomly overlapped with each other but yet homogenously distributed on the PPy/rGO layer. Adding to that, the flakes are in nanosized varying from 200 to 300 nm with obvious edges. The attachment of nanosized flakes on the PPy/rGO with uniform distribution facilitates the ion transport within the layers other than providing more active sites and channels for the electrolytes. The well-layered LBL film also helps in improving the surface area and concurrently enhanced the charge storage performance.

### 3.2 Fourier transform infrared spectroscopy

**Fig. 3:**
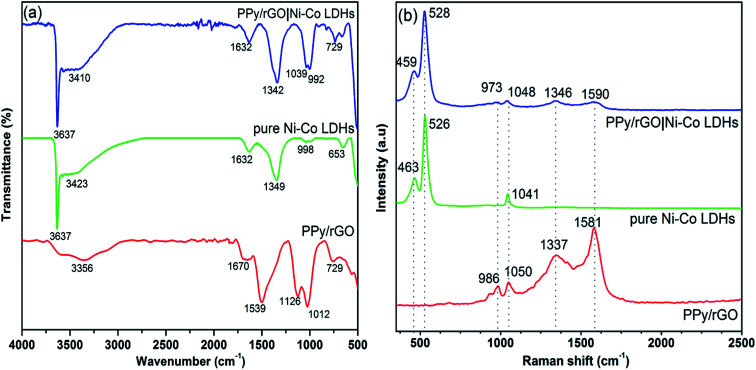
(a) FTIR and (b) Raman spectra of PPy/rGO, pure Ni–Co LDHs powder and PPy/rGO|Ni–Co LDHs LBL film.

FTIR measurements were conducted to demonstrate the formation of PPy/rGO with Ni–Co LDHs and the spectra of PPy/rGO, Ni–Co LDHs and LBL film are shown in Fig. 3a. In the PPy/rGO spectrum, the broad peak at 3356 cm^−1^ corresponds to O–H stretching vibration of the hydroxyl groups.^17^ Whereas, the characteristic peaks of PPy are also noticed at 1670, 1539 and 1126 cm^−1^, designated to C–C, C=C and C–N stretching vibrations, respectively.^18^ The peak observed at 1012 cm^−1^ represents the C–O–C stretching of epoxy group in rGO, while the polymerized pyrrole ring is revealed at 729 cm^−1^.^19^ As seen in the spectrum of Ni–Co LDHS, a sharp and narrow peak at 3637 cm^−1^ indicates the stretching of non-hydrogen bonded hydroxyl groups, and the broad peak at 3423 cm^−1^ is related to hydrogen bonded hydroxyl groups stretching vibration in LDHs.^20^ A band at 1632 cm^−1^ is identified as the O–H bending vibration of the adsorbed water molecules in the LDHs.^21^ Peaks at 1349 and 998 cm^−1^ correspond to the N–O (*υ*_3_ and bending) vibration of the adsorbed NO_3_^−^ ions (interlayer anion) which is from the precursor solution used in the preparation of LDHS.^20^ The peaks below 800 cm^−1^ are identified as the stretching and bending vibrations of the metal–oxygen (Ni–O–H and Co–O–H) in the hydrotalcite-like lattice.^22^ All the characteristic peaks of PPy/rGO and Ni–Co LDHs are noticed in the LBL film, indicating the PPy/rGO|Ni–Co LDHs composite is successfully prepared.

### 3.3 Raman spectroscopy

Structural interpretation of the composites was further studied *via* Raman spectra as displayed in Fig. 3b. PPy/rGO spectrum exhibits distinctive G-band (1581 cm^−1^) and D-band (1337 cm^−1^), signifying the sp^2^-graphitic structure due to E_2g_ phonon modes and defects presents in the rGO, respectively. Additionally, a peak at 1050 cm^−1^ manifesting C–H in-plane bending modes of PPy. More importantly, the obvious peak at 986 cm^−1^ with adjacent shoulder peak shows the deformation of the pyrrole ring, suggesting PPy is successfully introduced with rGO.^10^ The Raman spectrum of pure Ni–Co LDHs shows characteristic peaks at 463 and 526 cm^−1^ that related to Ni–O and Co–O vibrational modes in LDHs, respectively.^23^ The presence of these peaks validating the formation of LDHs.^24^ Whereas, the peak around 1041 cm^−1^ corresponds to residual nitrate anions.^23^ All the characteristic peaks of PPy/rGO and Ni–Co LDHs are observed in the LBL composite film. However, the PPy/rGO peaks in the LBL are less intense as the PPy/rGO is the bottom layer.

### 3.4 X-ray diffractometry

**Fig. 4:**
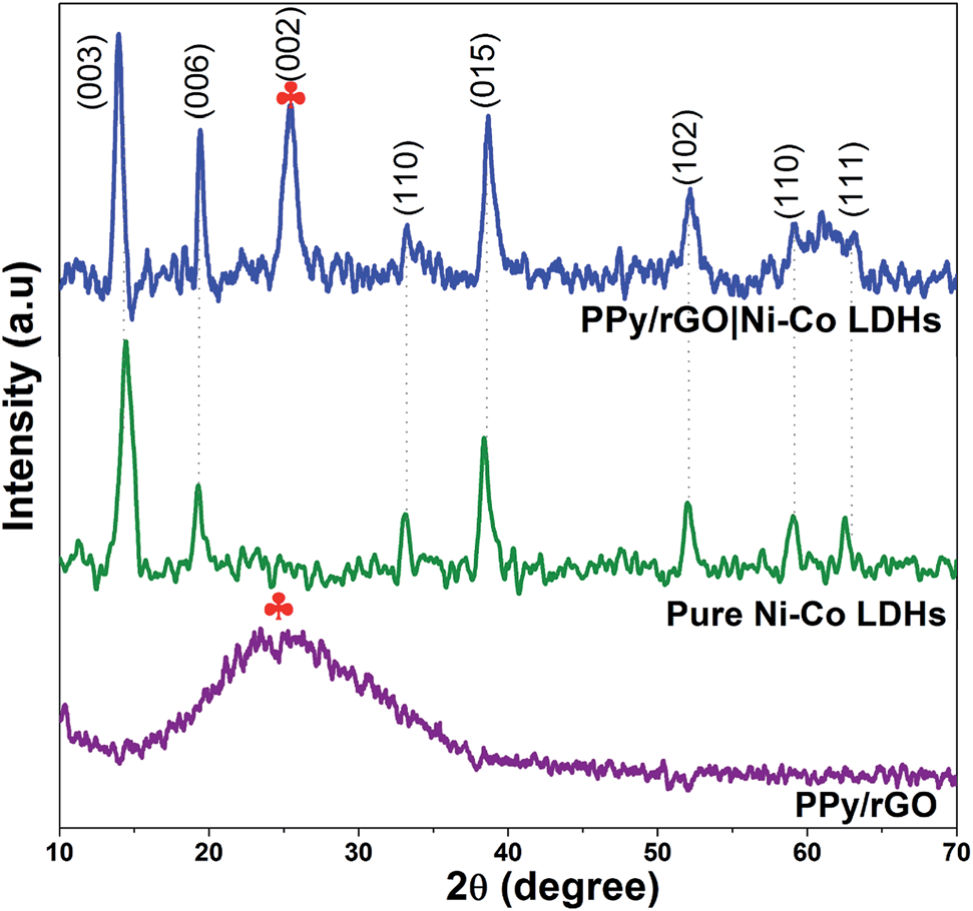
The XRD spectra of PPy/rGO, pure Ni–Co LDHs powder and PPy/rGO|Ni–Co LDHs LBL film.

XRD was used to evaluate the phase composition of the obtained samples. The XRD patterns of PPy/rGO, pure Ni–Co LDHs and LBL film are depicted in Fig. 4 to confirm the formation of LDHs and LBL assembled composite. As shown in the spectrum of PPy/rGO, it only displays a broad peak (labelled with 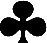) at 2*θ* = 25.2° (002). It can be interpreted as the amorphous nature of PPy.^15^ However, in the same position, the rGO also displays a diffraction peak related to the graphite-like structure composed of a few layers stacked graphene sheets.^25^ Therefore, it can be attributed to the diffraction pattern of either PPy or rGO. Whereas, the pure Ni–Co LDHs have few diffraction peaks at 2*θ* = 13.4°, 20.3°, 33.2°, 38.8°, 52.2°, 59.4° and 62.9° that can be indexed as (003), (006), (110), (015), (102), (110) and (111), respectively. These reflections of (003), (006) and (015) correspond to the lattice planes of hydrotalcite-like LDHs phase,^26,27^ thereby confirming the formation of LDHs. All the other diffraction peaks of Ni–Co LDHs are consistent with hexagonal phases of Ni(OH)_2_ (JCPDS card no. 003-0177) and Co(OH)_2_ (JCPDS card no. 045-0031). It is difficult to differentiate the phases between Ni(OH)_2_ and Co(OH)_2_ since their structures are similar and their diffraction peaks are close to each other. A combination of diffraction patterns of Ni–Co LDHs and PPy/rGO is seen in PPy/rGO|Ni–Co LDHs pattern, indicating the successful formation of the LBL composite film.

### 3.5 X-ray photoelectron spectroscopy

**Fig. 5:**
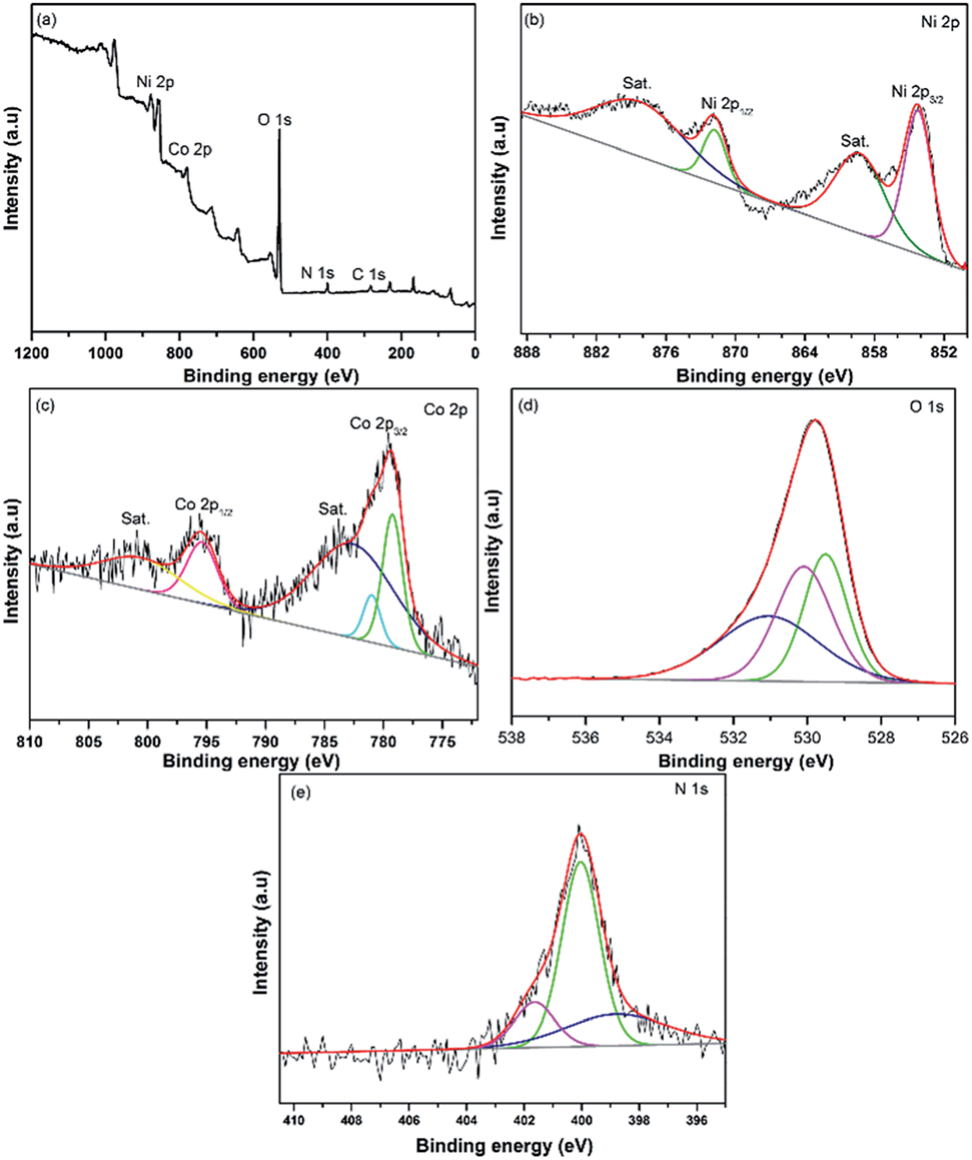
XPS spectra of PPy/rGO|Ni–Co LDHs LBL film (a) survey spectrum, (b) Ni 2p, (c) Co 2p, (d) O 1s and (e) N 1s.

XPS test was conducted to evaluate the valence state of the elements in the LBL assembled film. As shown in Fig. 5a, the main elements of the composite, Ni 2p, Co 2p, O 1s, C 1s and N 1s were identified. In the Ni 2p spectrum (Fig. 5b), four peaks were detected. The broad peaks at 859.6 and 878.4 eV correspond to the shake-up satellites (marked as Sat.). Whereas, the two main peaks of the composites located at 871.8 and 854.2 eV are attributed to the Ni 2p_1/2_ and Ni 2p_3/2_, respectively. In addition, the spin energy separation of 17.6 eV between the main peaks in the Ni 2p spectrum explaining the existence of valent state 2+ for nickel in the composite.^28,29^ Referring to the Co 2p spectrum (Fig. 5c), apart from the satellites peaks at 800.8 and 782.7 eV, the deconvoluted peaks at 795.4 and 781.1 eV are agreed with the valent state of cobalt species of 2+. While, the other fitting peak at 779.2 eV is ascribed to Co^3+^, revealing the co-existence of different valent states in the composite.^28,30^ The deconvoluted peaks in O 1s spectrum (Fig. 5d) show the bond between metal and oxygen (529.0 eV), the bond between metal and hydroxides (530.1 eV) and the absorption of oxygen (531.2 eV).^30^ The N 1s spectrum exhibits a major peak that can be deconvoluted into three main peaks ascribed to –N= (398.7 eV), –NH– (400.1 eV) and –N^+^– (401.65 eV).^31^

### 3.6 Cyclic voltammetry

**Fig. 6:**
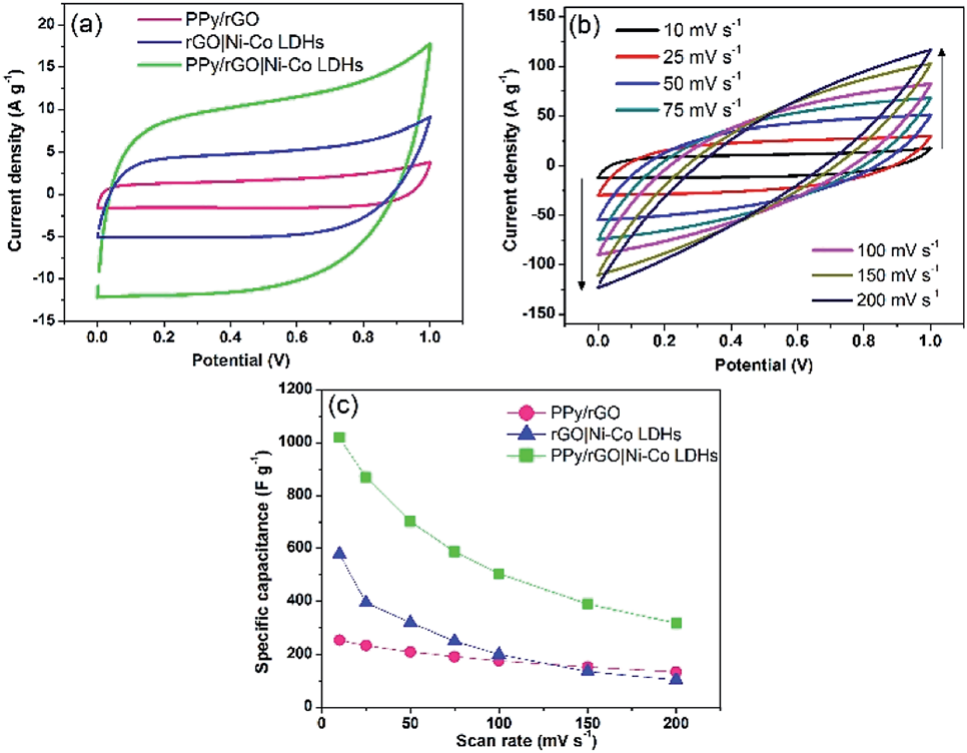
(a) A comparison CV curves of PPy/rGO, rGO|Ni–Co LDHs and PPy/rGO|Ni–Co LDHs LBL film at 10 mV s^−1^, (b) CV curves of PPy/rGO|Ni–Co LDHs LBL film at different scan rates and (c) comparison of specific capacitances of the symmetrical devices.

A two-electrode system was employed to study the electrochemical properties of the films for practical applications in 1 M Na_2_SO_4_. Fig. 6a displays the CV curves of PPy/rGO, rGO|Ni–Co LDHs and LBL film at 10 mV s^−1^. PPy/rGO exhibits a nearly rectangular shaped CV curve, whereas, at the same scan rate, the rGO|Ni–Co LDHs and LBL film show quasi-rectangular shape. In addition, the CV of LBL film has a larger loop area compared to PPy/rGO and rGO|Ni–Co LDHs, manifesting an increased electrochemical activity in the LBL film.^32^ This is due to the synergistic effects of each constituent from the formation of layers. The LBL film was further tested under different scan rates (Fig. 6b). Notably, the quasi-rectangular shape is slightly distorted from the lowest scan rate to the highest scan rate, implying the existence of internal resistance of the electrode material at high scan rates.^33^ Moreover, it shows that the layered structure is stable under various scan rates, benefiting from its intimate interaction between the layers. The *C*_sp_ values of the symmetrical electrode of PPy/rGO film and LBL film were calculated from the CV curve using eqn (1):1
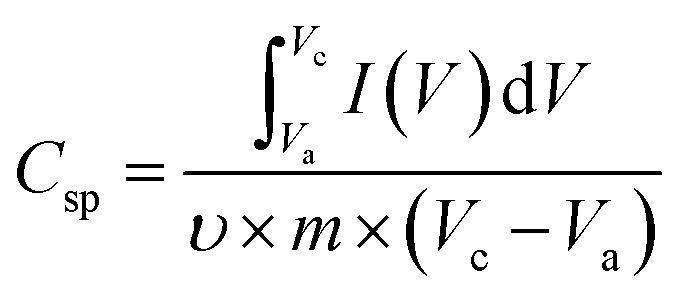
where *m* is the average mass of two electrodes (g), *V*_a_ and *V*_c_ are the integration limits of the CV curve (V), *I* is the response current (A), *υ* is the potential scan rate (V s^−1^), and *C*_sp_ is the specific capacitance (F g^−1^). At a scan rate of 10 mV s^−1^, the LBL film delivers *C*_sp_ of 1018 F g^−1^, which is four times higher than PPy/rGO and still able to deliver almost 400 F g^−1^ at a scan rate of 200 mV s^−1^ (Fig. 6c). The decrease in *C*_sp_ at higher scan rates could be the result of the high electrode resistance. Typically, at lower scan rates, the entire active area of the material is utilized by the ions from the electrolytes, thereby giving rise to the *C*_sp_. However, only the surface of the electrode material involved for the ion diffusion at higher scan rates, explaining the lower *C*_sp_.^34^ The obvious differences in *C*_sp_ between LBL film with PPy/rGO and rGO|Ni–Co LDHs can be attributed to the combination of pseudocapacitive and electrical double layer capacitive behavior in the LBL that further enhanced the supercapacitive performance. The overall enhanced performance of the LBL film could be ascribed to the direct formation of the electrode material without binder, the Ni–Co LDHs nanostructured layered firmly on PPy/rGO, generating abundant active sites for the ions from the electrolyte to diffuse easily and attachment of LDHs with PPy/rGO provides large accessible channels for electrolytes.

### 3.7 Galvanostatic charge/discharge

**Fig. 7:**
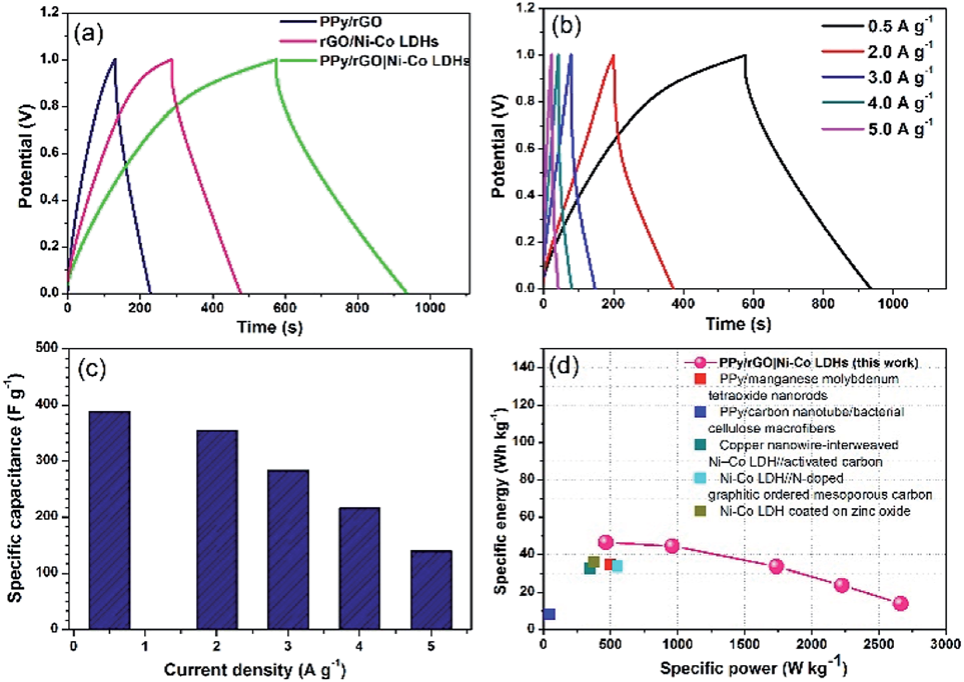
(a) A comparison GCD curves of PPy/rGO, rGO|Ni–Co LDHs and PPy/rGO|Ni–Co LDHs LBL film at 1 A g^−1^, (b) GCD curves, (c) comparison of specific capacitance of PPy/rGO|Ni–Co LDHs LBL film at different current densities and (d) Ragone plot of PPy/rGO|Ni–Co LDHs LBL film symmetrical device in comparison with literature.^1–5^

GCD measurements were also conducted within the same potential window as the CV measurements. As displayed in Fig. 7a, the PPy/rGO|Ni–Co LDHs LBL film exhibits a non-linear and asymmetrical charge–discharge curve signifying the pseudocapacitive performance of the electrode materials.^35^ Whereas, the PPy/rGO and rGO|Ni–Co LDHs films show a closely symmetrical charge discharge curves. Typically, compared with these charge discharge curves, the LBL film has the longest discharge time, demonstrating a better charge storage performance, which agrees with the CV results. However, a significant voltage drop (*IR* drop) is observed in the rGO|Ni–Co LDHs and LBL films at the initial part of the discharge curve, which is not seen in PPy/rGO, manifesting the presence of internal resistance in the former film.^36^ This result directly proportional to the equivalent series resistance (ESR) of the film, which will be further discussed in the EIS section. GCD measurements at different current densities as shown in Fig. 7b further reveal the good reversibility and capacitive performances of the LBL film. The charge–discharge duration of the film decreases as the current density increases. This characteristic indicates that at low current densities, the ions from the electrolyte have sufficient time to diffuse into the nanostructure of the active material. In the meantime, the inability of the ions to diffuse further deep in the active material and inaccessibility to the entire active material due to steric hindrance explaining the shortest discharge duration at high current densities.^37^

The *C*_sp_ values of the LBL film were calculated from the GCD curves (Fig. 7c) using eqn (2):2
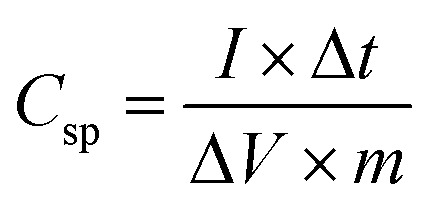
where Δ*t* is discharge time (s), *I* is the discharge current (A), and Δ*V* is the cell operation potential (V) and *m* is the average mass of two electrodes (g). The LBL film delivers a high *C*_sp_ value of 387 F g^−1^ at 0.5 A g^−1^. The values decrease steadily to 354, 283, 216 and 139 F g^−1^ when the current densities increase to 2, 3, 4 and 5 A g^−1^, respectively. At high current densities, the *IR* drop is high explaining the decrement in *C*_sp_ values.

Ragone plot is an important parameter to evaluate the performance of SCs. The specific energy, *E* (W h kg^−1^) and specific power, *P* (W kg^−1^) of the LBL film were calculated based on the following equations:3
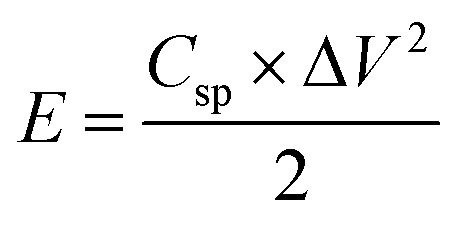
4
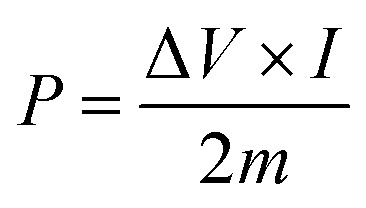
where *C*_sp_ is the specific capacitance (F g^−1^), *I* is the discharge current (A), and Δ*V* is the cell operation potential (V) and *m* is the average mass of two electrodes (g). As shown, the LBL film symmetrical device is capable of delivering a high *E* of 46.5 W h kg^−1^ at a *P* of 464.9 W kg^−1^ (Fig. 7d). These results are superior with other PPy- and asymmetrically assembled LDHs based electrode materials reported in the literature.^1–5^ All the above-mentioned results confirm the extraordinary performances of the LBL film and suitability as an electrode material for SCs.

### 3.8 Electrochemical impedance spectroscopy

**Fig. 8:**
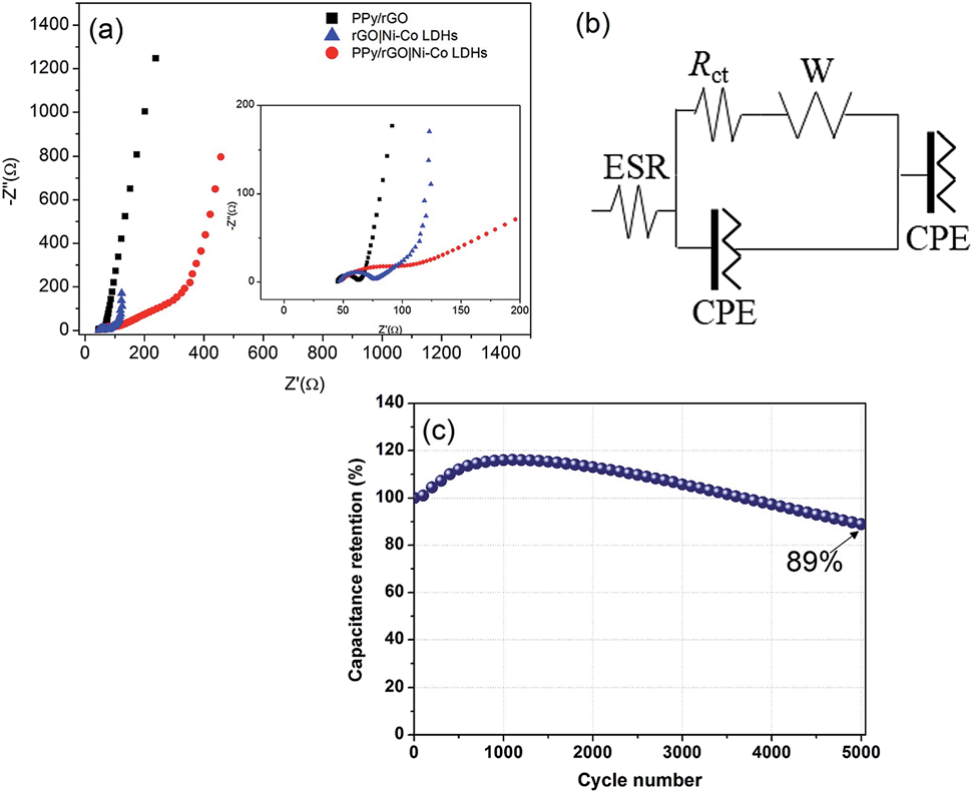
(a) Nyquist plot of PPy/rGO, rGO|Ni–Co LDHs and PPy/rGO|Ni–Co LDHs symmetrical electrode (inset shows magnification of Nyquist plot at high frequency), (b) the electrical equivalent circuit used to fit the Nyquist plots (c) the long term cycling performance of the PPy/rGO|Ni–Co LDHs symmetrical electrode at 200 mV s^−1^ over 5000 cycles.

With the interest of obtaining additional information on resistive and capacitive traits of the prepared films, EIS measurements were conducted and displayed in Fig. 8a. The resulting Nyquist plots exhibit a semicircle at high frequency region followed by a straight line at low frequency region. The semicircle is associated with the resistance of charge transfer (*R*_ct_) over the interface between electrolyte and electrode material,^38^ whereas a straight line demonstrating resistance of ionic transport, known as the Warburg diffusion line.^39^ An equivalent circuit is used to fit the obtained results as shown in Fig. 8b, including Warburg element (W), *R*_ct_, constant phase element (CPE) and ESR. The CPE is included in the circuit, replacing the double layer capacitance due to non-homogenous and irregular morphology as shown in FESEM micrographs. Furthermore, CPE represents the combination of pseudocapacitance and double layer capacitance of the system.^40^ In high frequency region of the Nyquist plot, the intercept point at *X*-axis represents the ESR. As shown in the inset of Fig. 8a, the ESR values of PPy/rGO, rGO|Ni–Co LDHs and LBL film are 44.9 Ω, 47.2 Ω and 49.1 Ω, respectively. The slight difference in the ESR value between both films could be ascribed to the presence of LDHs with low conductivity. Whereas, the LBL film exhibits a larger *R*_ct_ of 77.1 Ω than PPy/rGO (17.8 Ω) and rGO|Ni–Co LDHs (31.1 Ω), demonstrating the high interfacial resistance.^41^ Clearly, it shows that the LDHs in the LBL film is highly responsible for the *R*_ct_ of the system. Thus, it is further certified the observation from CV and GCD where the redox reaction certainly important for charge storage. Apart from this, the small nanoflake-like structure of LDHs in the LBL film as seen from FESEM in Fig. 2c, providing a larger electrode–electrolyte interface.

### 3.9 Cycling stability

Another essential interest in SC is long term cycling stability. The stability performance of the as-obtained LBL composite was measured over 5000 cycles at 200 mV s^−1^ as shown in Fig. 8c. It can be noticed that at the beginning of the cycling stability measurements, the capacitance retention rises to 116% during the charge–discharge cycles up to the 1200^th^ cycle. The initial enhancement in the retention can be ascribed to the self-activation process where the electrolyte gradually penetrating into the active material, resulting in the activation of redox species of the active materials.^42^ With the further increase in the number of cycles, the capacitance retention gradually decays to 89% of its initial capacitance after 5000 cycles. The major drawback of LDHs based electrodes is poor stability due to structural instability of LDHs during the continuous charge–discharge cycling process. The decay in the capacitance retention should be attributed to the structural instability of the LDHs, however, in this study, such low fading in the overall capacitance retention (11%) of the LBL film compared to other reported Ni–Co LDHs based asymmetrical electrodes such as Ni–Co–manganese LDHs/GO//activated carbon (63.3% retention over 5000 cycles),^43^ 3D-rivet graphene/Ni–Co LDHs//3D hierarchical graphene (80% retention over 10 000 cycles)^44^ and Ni–Co LDHs nanosheets//activated carbon (70.3% over 2000 cycles),^45^ explaining that PPy/rGO plays an important role. Layering Ni–Co LDHs on PPy/rGO enabling the composite film to adapt the volume expansion and contraction during continuous charge–discharge process benefiting from the high mechanical strength of PPy/rGO. The layered structure of the composite film also ensures the ion transport pathway is shortened, thus providing faster ions movement. Moreover, PPy/rGO with extraordinary mechanical strength provides extra support for the overall structure.

## 4. Conclusions

A novel free-standing film, PPy/rGO|Ni–Co LDHs was fabricated through a stepwise straightforward method utilizing the LBL approach. The high mechanical stability of PPy/rGO allows the formation of Ni–Co LDHs on the PPy/rGO. The symmetrical PPy/rGO|Ni–Co LDHs device delivered a high specific capacitance of 1018 F g^−1^ and high specific energy of 46.5 W h kg^−1^ at 464.9 W kg^−1^. The fabricated electrode material also possesses good cycling stability, 89% over 5000 continuous cycles. This is due to the large electroactive sites from LBL architecture and hexagonal flakes-like Ni–Co LDHs, high conductivity of rGO, high capacitance contribution of PPy and their synergy which led to enhanced structural stability.

## Conflicts of interest

There are no conflicts to declare.

## Supplementary Material
